# Ultraprotective ventilation allowed by extracorporeal CO_2_ removal improves the right ventricular function in acute respiratory distress syndrome patients: a quasi-experimental pilot study

**DOI:** 10.1186/s13613-020-00784-3

**Published:** 2021-01-07

**Authors:** Suzanne Goursaud, Xavier Valette, Julien Dupeyrat, Cédric Daubin, Damien du Cheyron

**Affiliations:** 1 CHU de Caen Normandie, Service de Réanimation Médicale, Av côte de Nacre, 14000 Caen, France; 2 Normandie Univ, UNICAEN, INSERM, U1237, PhIND “Physiopathology and Imaging of Neurological Disorders”, Institut Blood and Brain @ Caen-Normandie, Cyceron, 14000 Caen, France

**Keywords:** Acute respiratory distress syndrome, Extracorporeal CO_2_ removal, Protective mechanical ventilation, Right ventricular dysfunction, Critical care echocardiography

## Abstract

**Background:**

Right ventricular (RV) failure is a common complication in moderate-to-severe acute respiratory distress syndrome (ARDS). RV failure is exacerbated by hypercapnic acidosis and overdistension induced by mechanical ventilation. Veno-venous extracorporeal CO_2_ removal (ECCO_2_R) might allow ultraprotective ventilation with lower tidal volume (*V*_*T*_) and plateau pressure (*P*_plat_). This study investigated whether ECCO_2_R therapy could affect RV function.

**Methods:**

This was a quasi-experimental prospective observational pilot study performed in a French medical ICU. Patients with moderate-to-severe ARDS with PaO_2_/FiO_2_ ratio between 80 and 150 mmHg were enrolled. An ultraprotective ventilation strategy was used with *V*_*T*_ at 4 mL/kg of predicted body weight during the 24 h following the start of a low-flow ECCO_2_R device. RV function was assessed by transthoracic echocardiography (TTE) during the study protocol.

**Results:**

The efficacy of ECCO_2_R facilitated an ultraprotective strategy in all 18 patients included. We observed a significant improvement in RV systolic function parameters. Tricuspid annular plane systolic excursion (TAPSE) increased significantly under ultraprotective ventilation compared to baseline (from 22.8 to 25.4 mm; *p* < 0.05). Systolic excursion velocity (*S’* wave) also increased after the 1-day protocol (from 13.8 m/s to 15.1 m/s; *p* < 0.05). A significant improvement in the aortic velocity time integral (VTIAo) under ultraprotective ventilation settings was observed (*p* = 0.05). There were no significant differences in the values of systolic pulmonary arterial pressure (sPAP) and RV preload.

**Conclusion:**

Low-flow ECCO_2_R facilitates an ultraprotective ventilation strategy thatwould improve RV function in moderate-to-severe ARDS patients. Improvement in RV contractility appears to be mainly due to a decrease in intrathoracic pressure allowed by ultraprotective ventilation, rather than a reduction of PaCO_2_.

## Background

Although the current guidelines recommend a protective ventilation strategy [1] and adjuvant therapeutics such as prone positioning and neuromuscular blockade [2, 3], acute respiratory distress syndrome (ARDS) remains associated with significant mortality [4–6]. This is partly due to hyperinflation, which may promote ventilator-induced lung injury (VILI) [7–9]. It is now well established that pulmonary capillary lesions are associated with alveolar lesions, that specifically lead to pulmonary hypertension [10], worsened by local hypoxic vasoconstriction. Pulmonary hypertension may be associated with right ventricular (RV) dysfunction and/or acute cor pulmonale in 20 to 50% of patients with ARDS ventilated with a protective strategy [11]. Right ventricular failure has a deleterious impact on ARDS prognosis [12, 13], and experts recommend a systematic echocardiography assessment to evaluate RV function in patients with ARDS [14].

Many studies have shown that respiratory settings in ARDS patients are associated with RV function by several pathophysiological mechanisms. First, the deleterious effect of positive pressure correlated with tidal volume (*V*_*T*_) on the right ventricle is well demonstrated. A study showed that the incidence of RV failure was related to plateau pressure (*P*_Plat_) [12]. Second, the application of high positive end-expiratory pressure (PEEP), which is actually recommended in ARDS, may also overload the right ventricle [13, 15]. Third, it was suggested that decreased *V*_*T*_ and *P*_plat_ could decrease driving pressure, which was recently identified as a risk factor for mortality in ARDS [16]. However, this strategy can lead to hypercapnia and acidosis, which may worsen RV overload and ultimately RV function [17]. Therefore, it is now important to consider an RV-protective approach. The goal of this strategy is to limit *P*_Plat_, titrate PEEP, and ultimately limit hypercapnia. This theoretical approach to preserving RV function in ARDS patients is sometimes difficult to implement in clinical practice. Additional measures as prone positioning could improve RV function [18]. While no strong recommendations routinely support this device in ARDS, veno-venous extracorporeal CO_2_ removal (ECCO_2_R) is actually used to facilitate ultraprotective ventilation while avoiding risks of hypercapnia, acidosis and injurious ventilator settings [19–23]. Currently, main indications for ECCO_2_R in French intensive care units are ultraprotective ventilation for ARDS patients, shortening the duration of invasive mechanical ventilation in chronic obstructive pulmonary disease (COPD) patients, preventing intubation in COPD patients, and controlling hypercapnia and dynamic hyperinflation in mechanically ventilated patients with severe acute asthma [24, 25].

Despite an interesting physiological approach, no study has investigated the potentially beneficial role of ECCO_2_R in RV function. Therefore, the aim of the current quasi-experimental pilot study was to assess the impact of an ultraprotective ventilation strategy facilitated by low-flow veno-venous ECCO_2_R on RV function in patients with moderate-to-severe ARDS.

## Methods

### Study design and procedure

This quasi-experimental observational prospective pilot study was conducted between January 2017 and March 2019 in a French intensive care unit (ICU) of an academic hospital. The protocol was approved by appropriate legal and ethics authorities (Comité de Protection des Personnes Ile-de-France 6, Paris, France; no. 17032, June 29, 2017). Informed consent was obtained from patients or legally authorized surrogates.

### Patients

The inclusion criteria were as follows: severe-to-moderate ARDS with PaO_2_/FiO_2_ between 80–150 mmHg with fraction of inspired oxygen (FiO_2_) ≥ 60%; under protective invasive mechanical ventilation and sedation; and ARDS of pulmonary origin to have a homogeneous patient cohort. Exclusion criteria were age < 18 years, pregnancy, contraindication for systemic anticoagulation, platelet count < 50 Giga/L, moribund patients or those with treatment-limitation decisions, pre-existing severe treated pulmonary arterial hypertension, significant mitral valvulopathy, ARDS from extra-pulmonary cause and poor echogenicity, making the echocardiography assessment impossible.

### *ECCO*_*2*_*R system*

ECCO_2_R was provided by a low-flow CO_2_-removal device (Prismalung^®^; Gambro-Baxter) integrated into the renal replacement therapy (RRT) platform (PrismaFlex^®^; Gambro-Baxter). Of note, this device is currently unavailable, pending the new Prismalung Plus device (Gambro-Baxter). The polymethylpentene, hollow fiber, and gas-exchanger membrane (surface area 0.32 m^2^) was connected to the extracorporeal circuit. When RRT was required, both techniques were performed simultaneously. A 13 or 14-Fr double-lumen hemodialysis catheter (Gamcath^®^; Gambro-Baxter) was aseptically and percutaneously inserted under ultrasonography guidance into the internal jugular or femoral vein. Systemic anticoagulation was started after catheter insertion. We performed a priming with unfractionated heparin, and maintained systemic continued heparinization with the goal of anti-Xa between 0.3 and 0.7 UI/mL.

### Study protocol

**Fig. 1:**
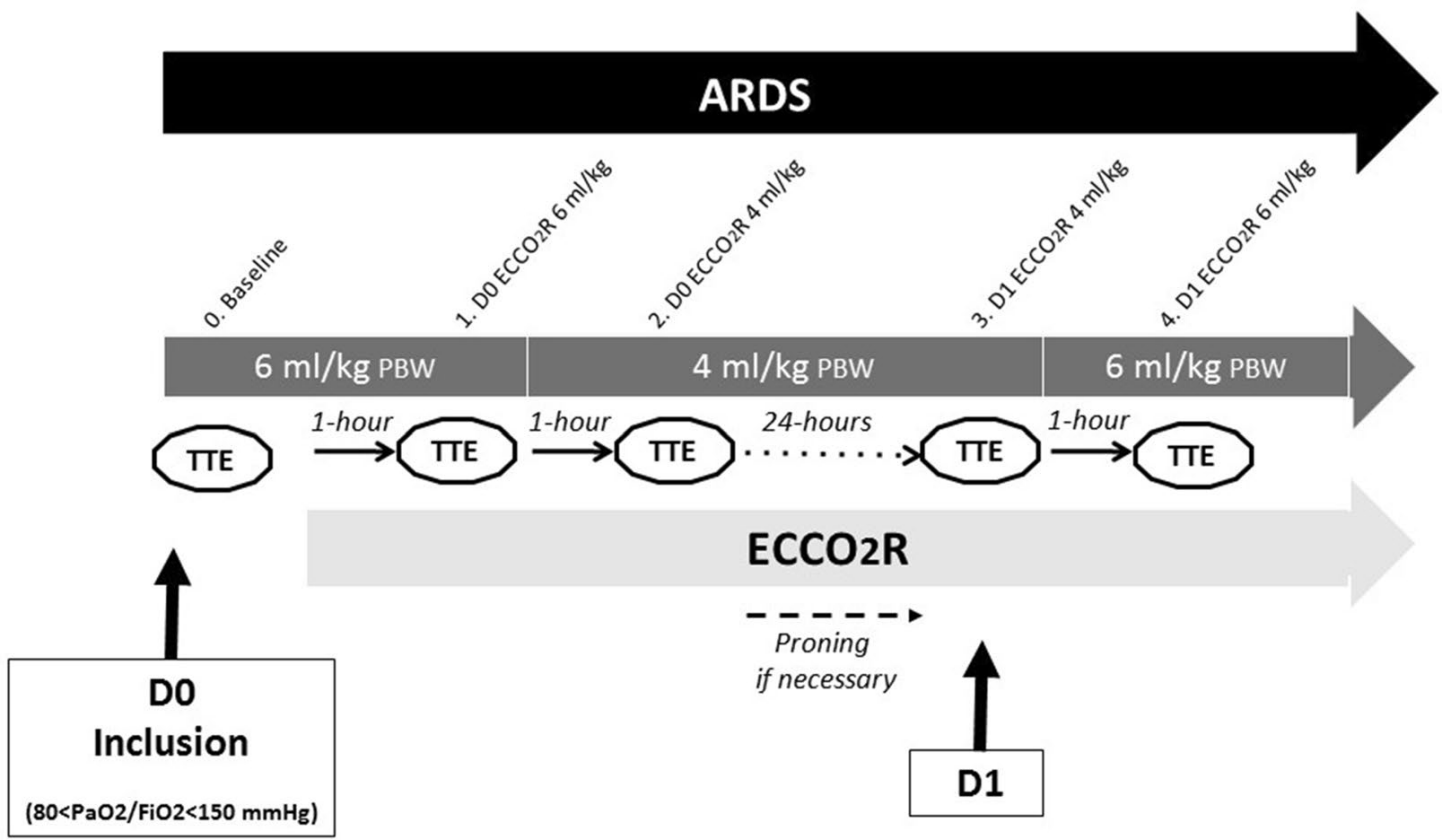
Study protocol. Transthoracic echocardiography (TTE), extracorporeal CO_2_ removal (ECCO_2_R), predictive body weight (PBW), day (D)

The study protocol is described in Fig. 1. At inclusion, patients were sedated, paralyzed, and ventilated in accordance with the recommendations for protective ventilation in ARDS [26]: *V*_*T*_ at 6 mL/kg of predicted body weight (PBW) and PEEP set to achieve a *P*_plat_ of 28–30 cm H_2_O. Transthoracic echocardiography (TTE) and blood gas were performed at inclusion. Then, after priming anticoagulation, the ECCO_2_R device was connected to the patient, and extracorporeal blood flow was progressively increased to 400 mL/min with sweep-gas flow through maintenance with 100% oxygen at 10 L/min during the entire protocol. After 1 h with ECCO_2_R, TTE and blood gas were performed simultaneously. Then, *V*_*T*_ was reduced from 6 to 4 mL/kg PBW under the ECCO_2_R device. After 1 h with these settings, TTE and blood gas were performed again. Then, the ECCO_2_R device was maintained for 24 h with ultraprotective ventilation parameters (4 mL/kg PBW). During the protocol, severe hypercapnia, acidosis and/or hypoxemia could be managed at the attending clinician’s discretion with changes in respiratory rate (RR) and *V*_*T*_ and with prone positioning and/or switching to ECMO if necessary. After 24 h under *V*_*T*_ 4 mL/kg PBW and ECCO_2_R, we also performed TTE and blood gas. After measurement, *V*_*T*_ was returned back to 6 mL/kg PBW with other settings adjusted according to the recommendations of protective ventilation. One hour later, under these standard conditions for the ventilatory management of patients with ARDS, TTE and blood gas were also performed. This last step allows the patient to be his or her own control in a quasi-experimental design. If, under these conditions, the patient remained stable, the ECCO_2_R device could be maintained after the protocol. The manufacturer determined the Prismalung^®^ membrane’s maximum duration to be 72 h.

### Data collection

Demographic data collected included age, gender, primary admission diagnosis, cause of ARDS, comorbidities and documented microorganisms during ARDS. Ventilator settings (*V*_*T*_, minute ventilation (VM), RR, PEEP, *P*_Plat_, FiO_2_, and driving pressure calculated as *P*_Plat_ minus PEEP [16], hemodynamic parameters, arterial blood gas values (partial alveolar oxygen pressure (PaO_2_), partial alveolar carbon dioxide pressure (PaCO_2_), bicarbonate (HCO_3_^−^), pH, lactate) were also collected. All TTE exams were made by two experienced physicians with specialized diploma in echocardiography to reduce the inter- and intra-observer variability. The TTE parameters evaluated were left ventricular ejection fraction (LVEF) at baseline, aortic velocity time integral (VTIAo), early diastolic mitral inflow velocity (*E*), atrial contraction mitral inflow velocity (*A*), E/A ratio, early diastolic mitral annular velocity (*E*’), RV systolic excursion velocity (*S*’), tricuspid annular plane systolic excursion (TAPSE), systolic pulmonary arterial pressure (sPAP), right/left ventricular end-diastolic diameter (RV/LV) ratio, and right atrial (RA) pressure estimation [27]. Heparin dose, anti-Xa, and ECCO_2_R parameters (blood flow, sweep-gas flow, characteristics of catheterization) were collected at baseline and then at the different times described previously. During the 24-h protocol, the need for prone positioning, nitric oxide, ECMO, and association with continuous RRT were recorded. Blood chemistry data and urine volume were collected daily. Physiologic data collected during and after the end of the protocol included the need for renal replacement therapy, ICU mortality, length of ICU stay, length of mechanical ventilation and duration under the ECCO_2_R system.

Patients were also monitored for severe adverse events until ICU discharge (clotting membrane, hemorrhagic complications, thrombocytopenia).

### Statistical analysis

The primary end point of the study was RV systolic function, assessed first by contractility parameters with TAPSE and S’ wave. Second, RV afterload (sPAP estimation), RV preload parameters (RV/LV ratio, RA pressure estimation, distensibility index), VTIAo (to assess cardiac output), and LV function (LVEF, LV end diastolic and systolic diameter, *E* velocity, *E/A* ratio, *E*’ velocity, *E/E’* ratio) were evaluated. The number of subjects required for the study was not calculated because of a pilot study design. Qualitative variables were expressed as numbers and percentages. Quantitative variables were expressed as the median and interquartile range (IQR; 25–75%). Changes in values are expressed as the percentage with the median and IQR. Comparisons between data over time during the protocol were performed using analysis of variance (ANOVA) for repeated measures. Missing data were integrated in the calculation. The comparison of one variable in 2 groups, taking into account (or correcting for) the variability of other variables (called covariates), was performed using analysis of covariance (ANCOVA). Values obtained at different times were compared with those obtained at baseline by using paired Student’s *t*-tests. The significance level was fixed at *p* < 0.05. Statistical analyses were performed using MedCalc Software (version 15.11.4).

## Results

Eighteen patients with moderate-to-severe ARDS were included in the study. The baseline characteristics of the patients at inclusion are shown in Table 1. The ECCO_2_R device was implemented in the early phase of ARDS (median time from intubation to ECCO_2_R start, 2 days). Neuromuscular blockade and prone positioning were applied before inclusion in 17 and 8 patients, respectively. In the 24 h following ECCO_2_R initiation, 6 patients received prone positioning. Four patients were under RRT at inclusion without additional patients thereafter. Two patients required vvECMO for worsening hypoxemia after the 24-h protocol. The operational characteristics of ECCO_2_R are summarized in Additional file 1: Table S1.

**Table 1:** Baseline characteristics of the 18 patients at study inclusion

Variables (units)	Median (IQR)
Sex (male/female)	13/5
Age (years)	64 (57–76)
Body mass index (kg/m^2^)	28 (24–31)
SAPS II at admission	42 (39–45)
SOFA score at ECCO_2_R insertion	6 (5–8)
PaO_2_/FiO_2_ (mmHg) at ECCO_2_R initiation	117 (100–136)
Comorbidities, *n* (%)	
Arterial hypertension	6 (33.3)
Chronic heart failure	6 (33.3)
Diabetes	5 (27.8)
Chronic renal impairment	2 (11.1)
Chronic obstructive pulmonary disease	0 (0)
Cause of ARDS, *n* (%)	
Pneumonia	15 (83.3)
Documented germs	12 (66.7)
Influenza	7 (38.9)
Others	5 (27.8)
Pulmonary contusion	1 (5.5)
Drug-induced pneumonia	1 (5.5)
Neoplastic lung disease	1 (5.5)
Pre-ECCO_2_R adjuvant therapy, *n* (%)	
Neuromuscular blockade	17 (94.4)
Prone positioning	8 (44.4)
Nitric oxide	0 (0)
Recruitment maneuvers	0 (0)
Time from ICU admission to ECCO_2_R initiation (days)	2.5 (1–4)
Time from intubation to ECCO_2_R initiation (days)	2 (1–3.8)

*ARDS* acute respiratory distress syndrome, *ECCO*_*2*_*R* extracorporeal CO_2_ removal, *SOFA* Sequential Organ Failure Assessment, *SAPS* Simplified Acute Physiologic Score, *IQR* interquartile range (25–75%)

**Fig. 2:**
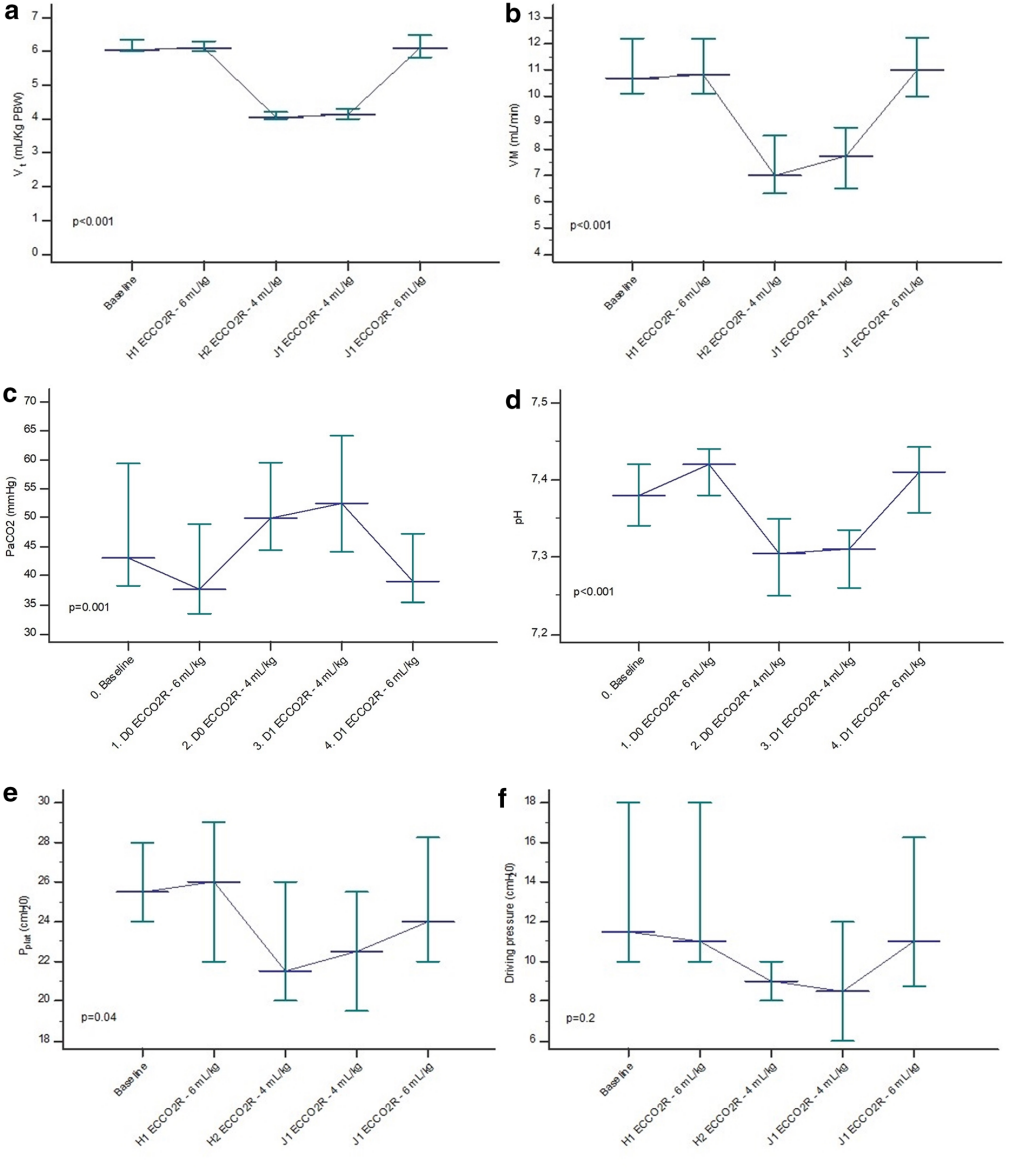
Time course of ventilation parameters during the study period. Tidal volume (*V*_*T*_) (**a**); minute ventilation (VM) (**b**); PaCO_2,_ partial alveolar carbon dioxide pressure (**c**); pH (**d**); plateau pressure (P_plat_) (**e**); driving pressure (DP) (**f**)

Ventilation settings, blood gas analysis, and renal and hemodynamic parameters throughout the protocol are shown in Table 2. The initial stepwise reduction *V*_*T*_ was significant in all 18 patients at a mean of 4.04 (4–4.19) mL/kg PBW one hour after CO_2_ removal began (*p* < 0.001). Initiation of ECCO_2_R resulted in a reduction in PaCO_2_ from 43.1 to 37.7 mmHg, a change of 13% from baseline (Fig. 2). There was a significant change in PaCO_2_ throughout the protocol (*p* = 0.001). Twenty-four hours after the ultraprotective ventilation strategy with ECCO_2_R, PaCO_2_ and pH were maintained at 52.5 mmHg (44.2–64) and 7.31 (7.27–7.33), respectively. The reduction in *V*_*T*_ was associated with a significant reduction in *P*_Plat_ from 25.5 (24–28) at baseline to 21.5 (20–25.8) cm H_2_O (D0 ECCO_2_R 4 ml/kg). The evolution of *P*_Plat_ remained significant throughout the study protocol (*p* = 0.04). *V*_*T*_ and *P*_Plat_ reduction with ECCO_2_R were not associated with a significant change in the PaO_2_/FiO_2_ ratio. All the renal and hemodynamic parameters did not differ significantly during ECCO_2_R therapy (Table 2).

**Table 2:** Ventilation, arterial blood gases, renal and hemodynamic parameters during the study period

	ECCO_2_R					Analysis of variance *p*
	*V*_*T*_ 6 mL/kg (n = 18)	*V*_*T*_ 6 mL/kg (n = 18)	*V*_*T*_ 4 mL/kg (n = 18)	*V*_*T*_ 4 mL/kg (n = 16)	*V*_*T*_ 6 mL/kg (n = 17)	
	Day 0		Day 1			
Ventilation variable						
*V*_*T*_ (mL/kg PBW)	6.06 (6–6.35)	6.11 (6–6.30)	4.04 (4–4.19)*	4.13 (4–4.28)*	6.09 (5.83–6.48)	< *0.001*
VM (L/min)	10.7 (10.1–12.2)	10.8 (10.1–12.2)	7 (6.4–8.4)*	7.8 (6.5–8.7)*	11 (10.1–12.2)	< *0.001*
RR (breaths/min)	28 (25–30)	28 (25–30)	29 (25–30)	30 (27.5–32)	28 (26–30)	*0.7*
PEEP (cm H_2_O)	11.5 (9.3–14.8)	11.5 (10–15)	12 (10–14.8)	13 (9.5–15.3)	13(10–16)	*0.9*
*P*_plat_ (cm H_2_O)	25.5 (24–28)	26 (22.3–28.8)	21.5 (20–25.8)*	22.5 (19.8–25.3)*	24 (22–28)	*0.04*
Driving pressure (cm H_2_O)	11.5 (10–17.8)	11 (10–17)	9 (8–10)	8.5 (6–11.5)	11 (9–16)	*0.2*
PaO_2_/FiO_2_ (mmHg)	108.5 (96.5–136.3)	109.5 (85.3–143)	116 (83.3–161)	113.5 (81.9–142)	112 (69–131)	*0.9*
Blood gases						
PaO_2_ (mmHg)	79.9 (68.3–88.7)	70.7 (64.7–87.8)	81.8 (70.7–110.4)	72.8 (68.3–95.1)	65.6 (58.3–79.4)*	*0.4*
PaCO_2_ (mmHg)	43.1 (38.2–57.9)	37.7 (33.5–48.3)*	50 (45.1–58.6)*	52.5 (44.2–64)*	39 (35.7–46.9)	*0.001*
Patients with PaCO_2_ > 50 mmHg at baseline (*n*, %)	7 (39)					
FiO_2_ (%)	70 (60–88.8)	70 (60–90)	80 (61.3–100)	85 (57.5–100)	75 (50–100)	*0.8*
pH	7.38 (7.34–7.42)	7.42 (7.38–7.44)*	7.31 (7.26–7.35)*	7.31 (7.27–7.33)*	7.41 (7.36–7.44)	< *0.001*
Lactate (mmol/l)	1.2 (0.9–1.4)	1.2 (1–1.7)	1.2 (0.9–1.4)	1.2 (1–1.5)	1.2 (1–1.6)	*0.6*
HCO_3_-(mmol/l)	23.6 (22.9–27.9)	22.6 (21.6–27.3)	23.4 (22.8–27.3)	25.2 (22.2–30)	24.9 (20.9–26.8)	*0.8*
Renal function						
Diuresis (ml)	860 (325–1191)		1700 (351–2050)			*0.06*
Creatinine (μmol/l)	72 (56–137)		81 (58–176)			*0.9*
Patients with CRRT (*n*, %)	4 (22)		4 (22)			*0.9*
Hemodynamic						
Mean arterial pressure (mmHg)	75 (70–80)	87 (79–95)*	84 (73–97)*	80 (73–87)	86 (68–87)*	*0.4*
Heart rate (beats/min)	84 (65–100)	75 (56–91)*	95 (57–100)*	86 (71–95)	76 (70–95)	*0.8*
Patients on norepinephrine, *n* (%)	4 (22.2)	4 (22.2)	2 (22.2)	4 (25)	5 (29.4)	
Norepinephrine dose (μg/kg/min)	0.25 (0.20–0.27)	0.21 (0.12–0.44)	0.11 (0.29–0.43)	0.24 (0.41–0.54)	0.19 (0.38–0.44)	*0.6*
Patients on dobutamine	1 (5.6)	1 (5.6)	1 (5.6)	1 (6.3)	1 (5.9)	
Total fluid resuscitation (ml)	250 (0–750)					
Prone positioning during protocol (*n*, %)	6 (33)					

Values presented as median and IQR, interquartile range (25–75%)

*PBW* predicted body weight, *ECCO*_*2*_*R* extracorporeal CO_2_ removal, *V*_*T*_, tidal volume, *VM* minute ventilation, *RR* respiratory rate, *PEEP* positive end-expiratory pressure, *P*_*plat*_ plateau pressure, *PaO*_*2*_ partial alveolar oxygen pressure, *FiO*_*2*_ fraction of inspired oxygen, *PaCO*_*2*_ partial alveolar carbon dioxide pressure, *HCO*_*3*_^*−*^ bicarbonate, *CRRT* continuous renal replacement therapy

*p < 0.05 vs baseline

**Fig. 3:**
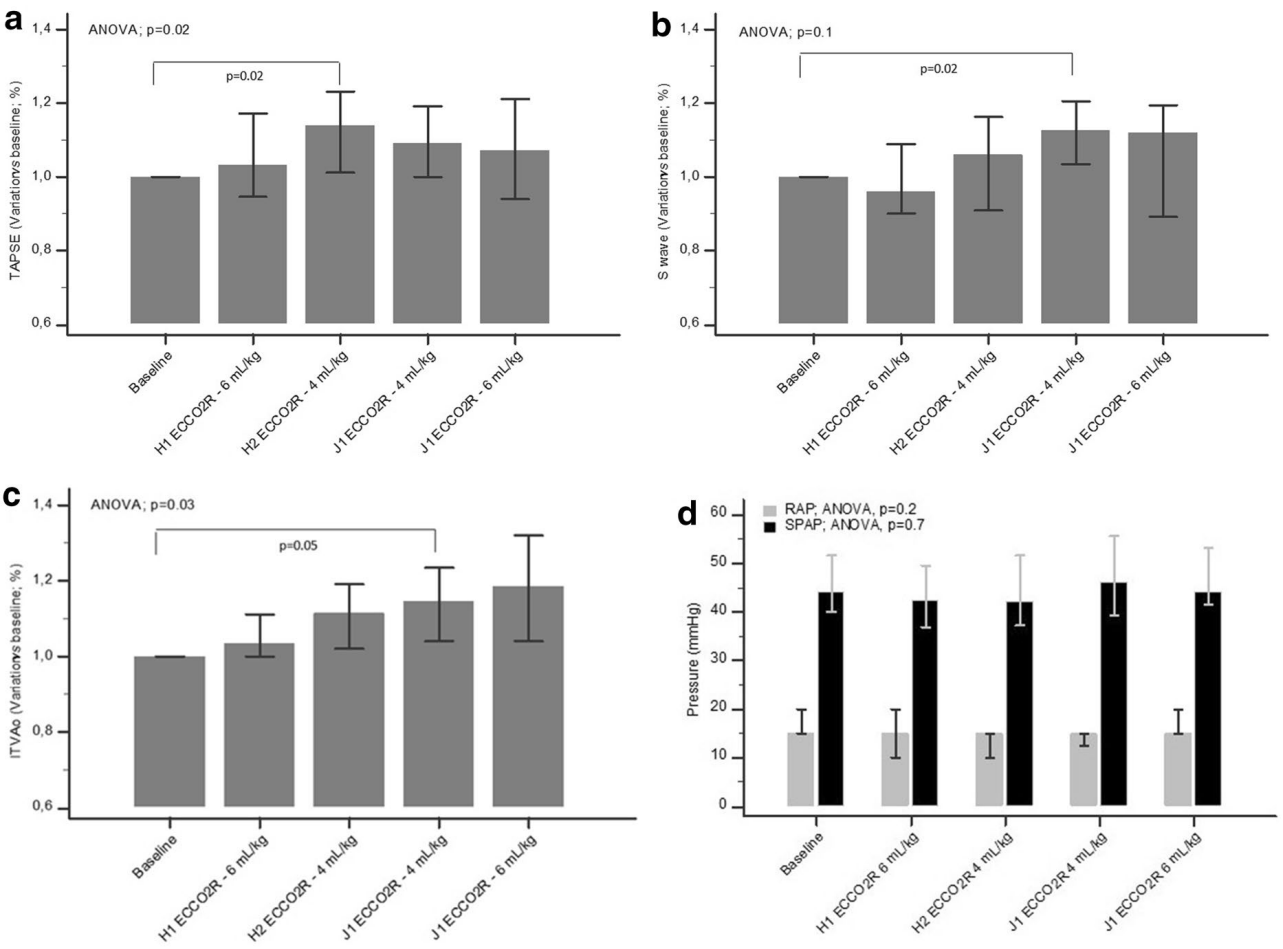
Time course of echocardiography variables during the study period. Tricuspid annular plane systolic excursion (TAPSE) (**a**); right ventricle systolic excursion velocity (*S*’) (**b**); aortic velocity time integral (VTIAo) (**c**); right atrial estimation pressure (RAP) and pulmonary systolic arterial pressure (sPAP) (**d**)

Echocardiographic characteristics are described in Table 3. Regarding RV systolic function, TAPSE changed significantly during the protocol with ECCO_2_R (*p* = 0.02), as illustrated in Fig. 3. More specifically, we observed a significant increase in TAPSE after initiation of the ultraprotective strategy with ECCO_2_R at day 0 (25.4 mm) compared to baseline (22.9 mm) (*p* = 0.02) (Additional file 1: Fig. S1). Individual data focused on RV parameters for patients who needed rescue therapies are given in Table 4. Assessment of correlation coefficient between TAPSE on the one hand, and *V*_*T*_ and PaCO_2_ on the other hand, found a significant (*r* = − 0.23; *p* = 0.03) and a non-significant (*r* = − 0.12; *p* = 0.26) inverse correlation for *V*_*T*_ and PaCO_2_, respectively. When we separated all 18 patients in two groups with normocapnia (PaCO_2_ < 50 mmHg; *n* = 11) and hypercapnia (PaCO_2_ > 50 mmHg; *n* = 7) at baseline, we observed a poorer TAPSE value in the hypercapnic patients than in the normocapnic patients but a significant improvement in TAPSE in both groups associated with low *V*_*T*_ at 4 mL/kg PBW (ANCOVA, *p* = 0.04) (Additional file 1: Fig. S2). S’ wave values did not change significantly overall throughout the study, but we observed a significant evolution between day 1, with a *V*_*T*_ reduced at 4 mL/kg PBW with ECCO_2_R, and the *S*’ wave median at baseline (15.1 versus 13.8 cm/s, respectively; *p* = 0.02). There was no significant difference in the other echocardiographic parameters reflecting RV function and RV preload. Regarding left ventricular systolic and diastolic function, parameters did not differ significantly throughout the study, whereas interestingly, we observed a significant increase in VTIAo values from 18.3 cm at baseline to 22.1 cm at day 1 ECCO_2_R 4 ml/kg (*p* = 0.05). Of note, RV variables of interest (TAPSE and *S’* wave velocity) returned at day 1 to a value close to the baseline value of day 0, after recovering standard conditions of ventilation with 6 mL/Kg *V*_*T*_.

**Table 3:** Time course of echocardiographic parameters during the study protocol

Parameter	ECCO_2_R					Analysis of variance* p*
	*V*_*T*_ 6 mL/kg (*n* = 18)	*V*_*T*_ 6 mL/kg (*n* = 18)	*V*_*T*_ 4 mL/kg (*n* = 18)	*V*_*T*_ 4 mL/kg (*n* = 16)	*V*_*T*_ 6 mL/kg (*n* = 17)	
	Day 0			Day 1		
Left ventricular function						
LVEF at baseline	57.5 (50–60.8)					
VTIAo (cm)	18.3 (15.7–22.1)	20.3 (16.6–22.9)	21.9 (16.2–24)	22.1 (19–25.4)*	20.8 (16.5–24.9)	*0.03*
LV end-diastolic diameter (cm)	4.8 (3.8–5.9)	4.7 (4–5.4)	4.8 (4–5.9)	4.4 (4–4.7)	4.5 (4–5.6)	*0.6*
LV end-systolic diameter(cm)	3.3 (2.9–4.2)	3.2 (2.8–4.3)	3.7 (2.9–4.2)	2.9 (2.8–3.5)	3.5 (2.8–4.1)	*0.6*
Left ventricular filling pressures estimation						
*E* velocity (m/s)	77.6 (61.4–91.5)	73.7 (64.9–101.3)	79.7 (69.8–100.8)	78.6 (66.9–95.4)	78 (63.6–96.6)	*0.9*
*E/A* ratio	1.02 (0.95–1.24)	1.04 (0.89–1.24)	1.11 (0.94–1.43)	1 (0.89–1.18)	0.98 (0.86–1.25)	*0.7*
*E*’ velocity (m/s)	8.43 (7.92–9.86)	10 (7.67–12.3)	9.72 (8.04–11.7)	9.63 (6.91–11.2)	9.32 (7.47–10.7)	*0.9*
*E/E*’ ratio	8.47 (7.28–11.05)	8.51 (7.28–11.3)	8.58 (6.74–11.12)	7.49 (7.15–9.47)	8.19 (7.13–9.88)	*0.9*
Right ventricular function						
* S*’ velocity (cm/s)	13.8 (12.5–15.3)	13.1 (12–14.2)	14.8 (12–15.9)	15.1 (14.3–17.2)*	14.7 (11.5–16.6)	*0.1*
TAPSE (mm)	22.9 (19.2–24.3)	25 (19.8–27)	25.4 (21.4–27.9)*	24.1 (21.4–26.9)	23.8 (20.8–26)	*0.02*
Paradoxical septal motion (*n*)	0	0	0	0	0	*–*
Tricuspid regurgitation gradient (mmHg)	27 (24–34)	29 (24–33)	33 (28–37)	30 (27–40)	29 (24–37)	*0.7*
sPAP estimation (mmHg)	44 (40–52)	43 (38–49)	42 (38–51)	46 (40–56)	44 (42–53)	*0.7*
Right ventricular preload						
RV/LV ratio	0.74 (0.57–0.79)	0.71 (0.61–0.77)	0.68 (0.62–0.74)	0.64 (0.59–0.81)	0.73 (0.61–0.79)	*0.8*
RA pressure estimation (mmHg)	15 (15–19)	15 (10–19)	15 (10–15)	15 (14–15)	15 (15–20)	*0.2*
Distensibility index	11.2 (5.2–20.8)	12.4 (5.4–25.1)	10.7 (6.8–22.3)	10.6 (6.5–16.9)	9.5 (6.1–14)	*0.6*

*LVEF* left ventricular ejection fraction, *VTIAo* aortic velocity time integral, E early diastolic mitral inflow velocity; E’ early diastolic septal mitral annular velocity, A: atrial contraction mitral inflow velocity, S’ right ventricle systolic excursion velocity, *TAPSE* tricuspid annular plane systolic excursion, sPAP systolic pulmonary arterial pressure, RV/LV ratio right/left ventricular end-diastolic diameter (RV/LV) ratio, RA right atrial

*p < *0.05 vs* baseline

**Table 4:** Individual ventilation parameters, RV echographic parameters and outcomes of patients who required rescue therapies

Patient	Etiology of ARDS	PaO2/FiO2 ratio at inclusion	Driving pressure at inclusion (cm H_2_O)	RV echocardiographic parameters										Pa O_2_ / FiO_2_ ratio before rescue therapy		Outcomes (at 28 days)
				TAPSE (mm)					*S’* velocity (cm/s)					Prone position	ECMO	
				*V*_*T*_ 6 mL/kg	*V*_*T*_ 6 mL/kg	*V*_*T*_ 4 mL/kg	*V*_*T*_ 4 mL/kg	*V*_*T*_ 6 mL/kg	*V*_*T*_ 6 mL/kg	*V*_*T*_ 6 mL/kg	*V*_*T*_ 4 mL/kg	*V*_*T*_ 4 mL/kg	*V*_*T*_ 6 mL/kg			
				ECCO_2_R					ECCO_2_R							
				DO			D1		DO			D1				
1	Toxic pneumonia	133	8	269	198	212	27	27	107	103	112	141	147			Alive
2	Documented bacterial pneumonia	137	17	245	26	28	21	242	154	139	151	18	12			Alive
3	Documented bacterial pneumonia	144	10	168	156	172	214	21	151	136	163	15	169			Alive
4	Non documented Interstitial pneumonia	132	8	254	25	25	248	237	136	131	148	162	16			Alive
5	Documented bacterial pneumonia (immunocompromised)	141	18	188	293	225	268	22	114	18	159	146	141			Alive
6	Toxic	134	19	28	288	351	333	254	127	142	135	141	109			Alive
7	Non documented pneumonia	98	10	204	198	224	–	194	143	12	12	–	115	80		Dead
8	Malignant hemopathy	143	19	122	168	15	–	178	82	91	118	–	953			Dead
9	Bacterial pneumonia	85	14	178	133	17	212	186	132	126	99	12	827	59		Alive
10	H1N1 pneumonia	90	10	231	286	285	27	293	117	133	14	143	151	91	80	Dead
11	H1N1 pneumonia	111	22	234	27	293	256	268	167	163	16	187	187			Dead
12	H1N1 pneumonia	105	11	261	27	274	268	24	128	113	10	144	141			Alive
13	H1N1 pneumonia	123	10	214	301	317	234	238	124	125	157	134	112			Alive
14	H1N1 pneumonia	106	10	231	–	266	234	204	175	–	138	169	223	158		Dead
15	H1N1 pneumonia	149	13	236	244	258	343	26	142	153	163	164	166		79	Dead
16	Toxic pneumonia	105	32	225	255	174	225	–	163	171	152	151	–	131		Dead
17	H1N1 pneumonia	86	12	186	173	22	199	285	172	103		185	167			Dead
18	Traumatic	93	9	226	214	258	214	208	139	13	16	18	147	111		Alive

Missing data are presented with endash (–)

Regarding the ECCO_2_R-related adverse events, we observed 5 (28%) membrane lung clotting events (Additional file 1: Table S2). Of note, 3 of the 5 clots of membrane were observed in the first patients included in the study, before HNF priming was increased from 25 to 50 UI/Kg. One severe adverse event occurred with a fatal intracerebral hemorrhage 24 h after the end of protocol, when the patient had been switched from the ECCO_2_R device to vvECMO because of worsening ARDS.

## Discussion

The results of this quasi-experimental pilot study showed that a low-flow ECCO_2_R device improves RV systolic function through the application of an early ultraprotective ventilation strategy, which reduces intrathoracic pressure during mechanical ventilation in moderate-to-severe ARDS patients. As previously described, the concept of the RV-protective approach was to limit *P*_Plat_ to decrease RV afterload [28]. On the other end, the RV approach also includes a limitation of hypercapnia. These two goals are allowed by the ECCO_2_R system. We could highlight a significant improvement in the RV contractility measured by TAPSE and *S*’ wave velocity associated with a reduction in tidal volume. From our study, it seems that improvement in RV contractility is mainly due to a “mechanic” effect with the decrease in intrathoracic pressure rather than a “metabolic” effect with PaCO_2_ and pH changes, but this question cannot be answered more precisely because of the study design and limitations. Interestingly, we showed that the improvement in RV systolic function is associated with increased VTIAo (cause or consequence; In fact, it is possible that changes in RV parameters we observed was related to increased VTIAo due to decreases in intrathoracic pressure and LV afterload [29]), without any changes in systolic or diastolic LV parameters. However, pathophysiology of RV function during ARDS with or without ECCO_2_R is complex, and many confounding factors (i.e., lung stress, PaCO_2_, pH, hemodynamics, etc.) could explain the observed variation of TAPSE, S’ wave velocity and VTIAo in our study.

Concerning the other RV parameters, we failed to highlight a significant modification of RV preload including RV/LV ratio, and afterload (sPAP) measures during the protocol period. RV function depends on preload, contractility and afterload, in particular through ventricular interdependence. No changes in RV preload (RA pressure estimation, distensibility index and RV dilatation) reflect constant and adequate volaemia. Consequently, our results support that RV preload did not affect directly change in TAPSE and VTIAo under ultraprotective ventilation, despite the lack of power of the study. Furthermore, no changes in RV afterload parameters suggest an improvement in RV contractility that arises with an ultraprotective ventilation strategy. However, our results for sPAP values should be treated with caution. Indeed, there is no specific agreement regarding the definition of pulmonary hypertension in ARDS. Using echocardiography, it is currently considered that a sPAP higher than 40 mmHg defines the presence of moderate pulmonary hypertension in ARDS [30]. The literature has shown a modest correlation between sPAP estimated from echocardiography and the gold-standard right-heart catheterization from mild-to-moderate pulmonary hypertension [31]. The sPAP could be influenced by several factors, such as hypercapnia, acidosis, hypoxia and mechanical ventilation. Moreover, applying high PEEP showed no effect in sPAP, while these maneuvres induced an increase in RV area and pulmonary vascular resistance [32]. The relevance of the sPAP measure compared to the RV function itself in estimations of the RV overload in ARDS is therefore questionable. Consequently, caution should be exercised in the interpretation of these findings.

In our study, we demonstrated the efficacy of a low-flow device to implement an ultraprotective ventilation strategy in ARDS patients, as previously described [33]. During these settings, the PaO_2_/FiO_2_ ratio remained consistent, and we did not have to increase the PEEP level. Some studies have suggested that lowering V_T_ may be associated with derecruitment [34]. Similar to previous report of *Allardet-Servent *et al*.* using the same RRT platform with an ECCO_2_R membrane [33], this was not the case in our study because our median PEEP levels close to 12 cmH_2_O were probably sufficient to prevent alveolar collapse.

The magnitude of the 13% PaCO_2_ reduction during the primary phase was lower than that observed in other studies [33, 35], which explains the gradual increase in PaCO_2_ during the V_T_ reduction phase at 4 mL/kg PBW. This statement is related to the low-flow device that we used compared with the high-flow devices which provide with higher CO_2_ extraction. However, in accordance with the recent literature, the V_T_ reduction phase at 4 ml/kg PBW was achievable in all cases [36], and hypercapnia and acidosis were not limiting factors during the protocol in our study. Because of the low level of decarboxylation allowed with a low-flow device ECCO_2_R, we were unable to decrease respiratory rate with the risk of worsen PaCO_2_ level and acidosis. Interestingly, we observed a deleterious effect of hypercapnia on RV function when we separated patients into normocapnic and hypercapnic patients with PaCO_2_ > 50 mmHg at baseline. However, early ultraprotective ventilation allowed by the ECCO_2_R device remains efficient on TAPSE in both groups.

The strengths of this study were its originality and design. To the best of our knowledge, no research has focused on the potential role of low-flow ECCO_2_R associated with ultraprotective ventilation on RV function despite a strong pathophysiological rationale. In ARDS, single-center or multicentre studies have examined the feasibility of ultraprotective ventilation facilitated by ECCO_2_R [21–23, 37, 38]. A case report described an improvement in RV function after one week of initiation of ECCO_2_R in a severe ARDS patient with an acute cor pulmonale [39]. Regarding preclinical research, a study examined the implementation of ECCO_2_R in a porcine model with experimental ARDS. The authors found a significant reduction in pulmonary arterial pressure [40]. However, it will be necessary to investigate more accurately on a larger scale, with multicentre studies, the potential place and utilization (i.e., timing) of ECCO_2_R to allow ultraprotective ventilation in the improvement of RV function and to what extent its potential benefits lead to improved prognosis in ARDS patients. Focusing on the same purpose, further larger studies will be necessary to specifically assess the safety and the benefit–cost ratio of the ECCO_2_R in the ARDS population.

Several limitations should be addressed in our study. First, because of the monocentric design and our small sample size (18 patients) which results in a lack of power, inferences from this quasi-experimental pilot study may be limited. Second, RV assessment with TTE could cause problems with reproducibility. The interrater reliability was not evaluated in our study, but previous studies found excellent results for right function valuation in echocardiography with low interobserver variability, particularly for the TAPSE [41]. Transoesophageal echocardiography (TEE) has been shown to be superior to TTE in diagnosing RV dysfunction in mechanically ventilated patients with moderate-to-severe ARDS [42], but is a more invasive tool. Third, in our study, the baseline values of TAPSE and *S*’ velocity were 22.9 mm and 13.8 cm/s, respectively, which were much higher than the threshold values under which RV systolic dysfunction is usually defined (16 mm and 10 cm/s, respectively) [41]. In addition, change in TAPSE values were not very impressive, and the values change from a normal to a supra normal value. Thus, assessment of TAPSE with ECCO_2_R in ARDS patients with RV dysfunction are now warranted. Moreover, our evaluation of RV function was not exhaustive. Other parameters, such as the right ventricular–vascular coupling (TAPSE/sPAP), or RV area, and RV wall thickness and RV outflow tract acceleration time, could also be measured. Similarly, for ventilator parameters, mean airway pressure was not monitored throughout the study protocol. Indeed, in accordance with the literature about ventilator factors influencing RV dysfunction, we focused our data collection on P_Plat_, driving pressure, V_T_ and PEEP. Fourth, concerning our population study, we made the choice to exclude extra-pulmonary ARDS in order to have a small homogeneous group of ARDS patients. Our choice could be discussed because of in contrast to previous study [43], recent meta-analysis and large cohort study showed no difference about the prognosis between pulmonary and extra-pulmonary ARDS patients [44, 45]. Finally, there was a high degree of heterogeneity in the severity of our ARDS patients. Almost one-third of patients have engaged in prone positioning sessions during the protocol. This should be considered as an important confounding factor because it may significantly improve RV function [18].

## Conclusion

This quasi-experimental study demonstrated that ultraprotective ventilation strategy facilitated by a low-flow ECCO_2_R device could improve RV systolic function in moderate-to-severe ARDS patients. Similarly to prone positioning, ECCO_2_R could become a strategy that enables the reconciliation of a lung protective approach with an RV-protective approach in ARDS patients. Large-scale clinical studies, including patients with severe RV dysfunction, are warranted to confirm these preliminary results and to assess the overall benefits of ECCO_2_R ARDS patients.

## Supplementary Information

**Additional file 1: Table S1. **Operational characteristics of extracorporeal CO_2_ removal during the study period for the 18 patients.** Table S2. **Adverse events and outcomes of the 18 patients receiving ECCO_2_R.** Figure S1.** Individual changes in TAPSE between baseline and 1-hour after reduction of VT at 4 mL/kg PBW with ECCO_2_R. Tricuspid annular plane systolic excursion (TAPSE) increased from 22.9 mm (19.2–24.3) to 25.4 mm (21.4–27.9) (p=0.02).** Figure S2.** Time course of TAPSE in two groups of patients with PaCO_2_ < 50 mmHg (n=11) and PaCO_2_ > 50 mmHg (n = 7) at baseline (ANCOVA, p = 0.04).

## Data Availability

All data generated and analyzed during this study are not publicly available due to lack of consent of the participants for dataset publication, but can be made available by the corresponding author upon reasonable request.

## Abbreviations

A: Atrial contraction mitral inflow velocity
ARDS: Acute respiratory distress syndrome
COPD: Chronic obstructive pulmonary disease
CRRT: Continuous renal replacement therapy
D: Day
E: Early diastolic mitral inflow velocity
E’: Early diastolic mitral annular velocity
ECCO_2_R: Extracorporeal CO_2_ removal
FiO_2_: Fraction of inspired oxygen
ICU: Intensive care unit
IQR: Interquartile range (25–75%)
LVEF: Left ventricular ejection fraction
NIV: Non-invasive ventilation
PaCO_2_: Partial alveolar carbon dioxide pressure
PaO_2_: Partial alveolar oxygen pressure
PBW: Predicted body weight
PEEP: Positive end-expiratory pressure;
P_Plat_: Plateau pressure
RA: Right atrial
RR: Respiratory rate
RRT: Renal replacement therapy
RV: Right ventricular
RV/LV ratio: Right/left ventricular end-diastolic diameter
*S’*: RV systolic excursion velocity
SAPS: Simplified acute physiologic score
SOFA: Sequential organ failure assessment
sPAP: Systolic pulmonary arterial pressure
TAPSE: Tricuspid annular plane systolic excursion
TEE: Transoesophageal echocardiography
TTE: Transthoracic echocardiography
vvECMO: Veno-venous extracorporeal membrane oxygenation
VILI: Ventilator-induced lung injury
VM: Minute ventilation
*V*_*T*_: Tidal volume
VTIAo: Aortic velocity time integral.
