# Supplementary material for: Ultraprotective ventilation allowed by extracorporeal CO2 removal improves the right ventricular function in acute respiratory distress syndrome patients: a quasi-experimental pilot study
Source: Ann Intensive Care. 2021 Jan 7;11:3. doi: 10.1186/s13613-020-00784-3 (PMC7788545; doi:10.1186/s13613-020-00784-3)

**Additional files**

**Ultraprotective ventilation allowed by extracorporeal CO2 removal improves the right ventricular function in ARDS patients: a quasi-experimental pilot study.**

Suzanne Goursaud^1,2^, Xavier Valette^1^, Julien Dupeyrat^1^, Cédric Daubin^1^, Damien du Cheyron^1^

1. CHU de Caen Normandie, Service de Réanimation Médicale, 14000 Caen, France.
2. Normandie Univ, UNICAEN, INSERM, U1237, PhIND « Physiopathology and imaging of Neurological Disorders », Institut Blood and Brain @ Caen-Normandie, Cyceron, 14000 Caen, France.

**Table S1. Operational characteristics of extracorporeal CO_2_ removal during the study period for the 18 patients.**

| Parameter | Baseline | ECCO_2_R | | | | | |
| --- | --- | --- | --- | --- | --- | --- | --- |
|  | **V_T_ 6 mL/kg**  (n=18) | | **V_T_ 6 mL/kg**  (n=18) | **V_T_ 4 mL/kg**  (n=18) | | **V_T_ 4 mL/kg**  (n=16) | **V_T_ 6 mL/kg**  (n=17) |
|  | Day 0 | | | | Day 1 | | |
| Blood flow (mL/min) | NA | | 400 (400-400) | 400 (400-400) | | 400 (400-400) | 400 (400-400) |
| Sweep gas flow (L/min) | NA | | 10 (10-10) | 10 (10-10) | | 10 (10-10) | 10 (10-10) |
| Time of utilization (hours) | 34.5 (24.5-49.5) | | | | | | |
| Heparin bolus at ECCO_2_R starting (IU/kg) | 87 (68-97) |  | | | | | |
| Heparin (IU/kg/day) | 408 (348-494) | | | | 348 (275-400) | | |
| AntiXa activity (UI/ml) | 0.16 (0.12-0.19) | 0.75 (0.48-1.30) | | | 0.54 (0.45-0.81) | | |

Values are expressed as median (IQR, interquartile range (25-75%)).

**Table S2. Adverse events and outcomes of the 18 patients receiving ECCO_2_R**

| ECCO_2_R-related adverse events | n (%) |
| --- | --- |
| Membrane lung clotting , n (%) | 5 (28) |
| Bleeding |  |
| At cannula insertion, n (%) | 0 |
| Significant, n (%) | 1 (6) |
| Infectious complication, n (%) | 0 |
| Thrombocytopénia, n (%) | 0 |
| Clinical outcomes |  |
| Death in ICU (n, %) | 8 (44) |
| 28-day mortality, n (%) | 8 (44) |
| Lenght of stay in ICU (days) | 15 (11-20) |
| Lenght of stay in ICU after inclusion (days) | 11 (7-17) |
| Duration of mechanical ventilation in ICU survivors (days) | 12 (8.3-15.8) |
| Duration of mechanical ventilation after inclusion in ICU survivors (days) | 7.5 (6-12) |
| SOFA score with ECCO_2_R | 8 (5-9) |
| Duration of catecholamines (days) | 3 (1.3-4) |
| Duration of catecholamine after inclusion (days) | 2 (0-3) |
| Patient requiring RRT after inclusion, n (%) | 3 (17) |
| RRT at ICU discharge, n (%) | 2 (11) |
| Creatinine level at ICU discharge (μmol/l) | 59 (38-101) |

ECCO_2_R, extracorporeal CO_2_ removal; ICU, Intensive care unit; SOFA, sequential organ failure assessment; RRT, renal replacement therapy.

Values are expressed as number (percentage) or median (IQR, interquartile range (25-75%)).

**Figure S1:** Individual changes in TAPSE between baseline and 1-hour after reduction of V_T_ at 4 mL/kg PBW with ECCO_2_R. Tricuspid annular plane systolic excursion (TAPSE) increased from 22.9 mm (19.2-24.3) to 25.4 mm (21.4-27.9) (*p*=0.02).

**
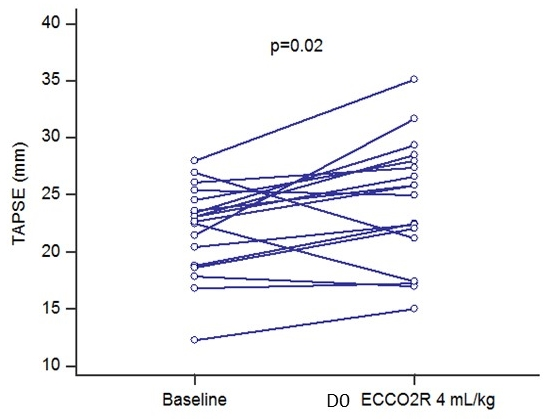
**

**Figure S2**: Time course of TAPSE in two groups of patients with PaCO_2_<50 mmHg (n=11) and PaCO_2_>50 mmHg (n=7) at baseline (ANCOVA, *p*=0.04).


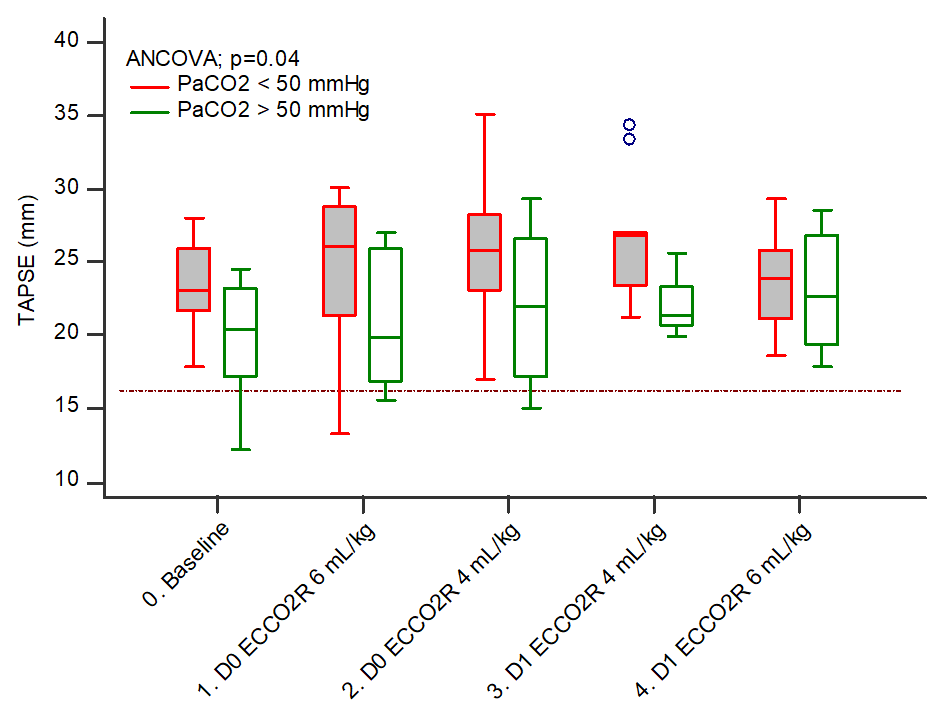

Supplement: Supplementary file 1 — Additional file 1: Table S1. Operational characteristics of extracorporeal CO2 removal during the study period for the 18 patients. Table S2. Adverse events and outcomes of the 18 patients receiving ECCO2R. Figure S1. Individual changes in TAPSE between baseline and 1-hour after reduction of VT at 4 mL/kg PBW with ECCO2R. Tricuspid annular plane systolic excursion (TAPSE) increased from 22.9 mm (19.2–24.3) to 25.4 mm (21.4–27.9) (p=0.02). Figure S2. Time course of TAPSE in two groups of patients with PaCO2 < 50 mmHg (n=11) and PaCO2 > 50 mmHg (n = 7) at baseline (ANCOVA, p = 0.04). [file 13613_2020_784_MOESM1_ESM.docx]
